# Flexible Lab-Tailored Cut-Offs for Suitability of Formalin-Fixed Tumor Samples for Diagnostic Mutational Analyses

**DOI:** 10.1371/journal.pone.0121815

**Published:** 2015-04-06

**Authors:** Sara Mariani, Cristiana Di Bello, Lisa Bonello, Fabrizio Tondat, Donatella Pacchioni, Luca Molinaro, Antonella Barreca, Luigia Macrì, Luigi Chiusa, Paola Francia di Celle, Paola Cassoni, Anna Sapino

**Affiliations:** 1 Department of Medical Sciences; University of Torino, Torino, Italy; 2 Azienda Ospedaliera Universitaria Città della Salute e della Scienza; Presidio Ospedaliero Molinette of Torino, Torino, Italy; 3 Department of Molecular Biotechnology and Health Sciences, University of Torino, Torino, Italy; University of Texas MD Anderson Cancer Center, UNITED STATES

## Abstract

The selection of proper tissues from formalin-fixed and paraffin-embedded tumors before diagnostic molecular testing is responsibility of the pathologist and represents a crucial step to produce reliable test results. The international guidelines suggest two cut-offs, one for the percentage and one for the number of tumor cells, in order to enrich the tumor content before DNA extraction. The aim of the present work was two-fold: to evaluate to what extent a low percentage or absolute number of tumor cells can be qualified for somatic mutation testing; and to determine how assay sensitivities can guide pathologists towards a better definition of morphology-based adequacy cut-offs. We tested 1797 tumor specimens from melanomas, colorectal and lung adenocarcinomas. Respectively, their BRAF, K-RAS and EGFR genes were analyzed at specific exons by mutation-enriched PCR, pyrosequencing, direct sequencing and real-time PCR methods. We demonstrate that poorly cellular specimens do not modify the frequency distribution of either mutated or wild-type DNA samples nor that of specific mutations. This observation suggests that currently recommended cut-offs for adequacy of specimens to be processed for molecular assays seem to be too much stringent in a laboratory context that performs highly sensitive routine analytical methods. In conclusion, new cut-offs are needed based on test sensitivities and documented tumor heterogeneity.

## Introduction

The identification of biologically active genes and pathways that are disrupted in various cancer types has led to the development of clinically relevant diagnostic requirements. Several oncogenes have become therapeutic targets [1–3]. Thus, oncology is rapidly moving towards an era of precision medicine, where patients receive tailored treatments based on the biology and the genetic makeup of their tumors [4]. In the last few years, the Food and Drugs Administration (FDA), the European Medicines Agency (EMA) and the Italian Medicines Agency (AIFA) have introduced many biological drugs in therapeutic schemes of various cancers. Among others, anti-epidermal growth factor receptor (anti-EGFR) monoclonal antibodies (Cetuximab and Panitumumab) were approved for the treatment of wild-type RAS metastatic colorectal cancers (mCRCs) and anti-tyrosine kinases (Erlotinib and Vemurafenib) were admitted in therapeutic schemes to treat EGFR mutated non-small cell lung cancers (NSCLCs) and BRAF-mutated metastatic melanomas (mMELs), respectively. The conditions of their clinical uses were determined by specific trials (CRYSTAL, IPASS, BRIM-1, PEAK and PRIME), designed on the basis of results from retrospective studies [5–8].

The development of such targeted therapies has contributed to establishing a strong collaboration between pathologists and molecular biologists to provide clinicians with the most accurate results. The histo-morphological revision and the selection of formalin-fixed and paraffin-embedded (FFPE) tumor tissues are essential prerequisites of the analytical phase to allow discrimination of patient eligibility for targeted therapies. The Italian guidelines suggest that for mutational analyses tissue samples should contain an absolute number of at least 100 tumor cells and a tumor cell percentage representation over 50% (*https://testbiomolecolari.it/*). The suitability of tumor specimens with low percentage and/or absolute number of cancer cells represents a grey zone for molecular analyses, still debated within the scientific community [9–12]. The present work focuses on DNA sequencing analyses applied to patient specimens with a poor tumor cellularity. The frequency distributions of mutated and wild-type DNA sequences in hotspot regions of K-RAS, BRAF and EGFR genes were compared in a large series of morphologically acceptable and not acceptable samples in order to understand the relationship between morphology-driven tissue selection and the pertinence to molecular biology analyses in the era of highly sensitive sequencing methods.

## Materials and Methods

### Patients

This study was performed on FFPE tumor samples subjected to DNA extraction and sequencing. Tissue specimens from oncologic patients were collected at our institution, which is one of the regional reference laboratories for the study of somatic mutations as predictive markers of response to targeted therapy-based regimens. The case series included the tumor tissues of 1084 mCRCs, 572 never-, low- or ex-smoking NSCLC patients at stages IIIb and IV, and 141 mMELs or inoperable melanomas at stages IIIc and IV. Cytological specimens and histological surgical samples were 190 and 1607, respectively. Fine-needle aspiration biopsies (FNAB) and brushing techniques were the most commonly used methods for cytological sampling. The paraffin-embedded samples had been stored for a variable period of time (from few months up to eight years) before molecular analyses were performed.

The study was submitted to and approved by the ethic institutional review board for "Biobanking and use of human tissues for experimental studies" of the Pathology Service of the Azienda Ospedaliera Universitaria Città della Salute e della Scienza di Torino, Torino, Italy. The project provided a verbal and not written informed consent from patients due to the retrospective approach of the study, which did not impact on their treatment. All the cases were anonymously recorded. The IRB approved this consent procedure.

### Material selection criteria

The selection of the tumor area was performed on Hematoxylin and Eosin (H&E) archival slides originally used for cytological examinations or histo-pathological diagnosis, classification and grading, according to World Health Organization criteria. On the basis of clonal evolution trends, primitive or relapsed tumors or synchronous distant metastases were preferred in mCRCs, NSCLCs and mMELs [13–15]. The pathologist marked the areas of tumor enrichment on the slide from either cytological or histological specimens and recorded the percentage and absolute number of cancer cells in the request form (see below). Accordingly, five serial sections were cut from the ethanol-fixed or FFPE blocks. The first four sections were 10 μm-thick and were collected in two sterile tubes (Eppendorf, Milano, Italy) for DNA extraction. The last section, 3 μm-thick and H&E-stained, was used to confirm the correspondence with the original marked areas and to further check the presence of tumor cells in the samples.

The criteria of morphological suitability for performing DNA mutational analyses were derived from the Biogate portal (*https://testbiomolecolari.it/*) of the Association of Italian Medical Oncologists (AIOM). In particular, specimens satisfying the guideline criteria were recorded as acceptable (*A*), whereas specimens submitted to tumor enrichment but with less than 50% (70% until 2011) or 100 tumor cells had to be considered as not acceptable (*NA*). We named these specimens *NA1* and *NA2*, respectively.

### DNA extraction

Enriched tumor sections from *NA* and *A* specimens were submitted to DNA extraction (Maxwell16 FFPE Tissue LEV DNA Purification Kit, Promega, Madison, WI, USA) on a Maxwell16 Instrument (Promega, Madison, WI, USA). DNA was eluted in a final volume of 50 μL, and the concentrations/purity was measured by a Nanodrop 1000 spectrophotometer (Thermo Fisher Scientific, Wilmington, DE, USA).

### Restriction enzyme-mediated selective polymerase chain reaction (REMS-PCR) for the analysis of the K-RAS gene mutations in codon 12

The K-RAS procedure was optimized by Fuery et al. [16]. Due to its high sensitivity, close to 0.1%, the assay lets to investigate nucleotide substitutions affecting even low copies of genomic DNA at the codon 12. REMS-PCR was assessed as previously described [17] with 120 ng of patient and control DNA in a final volume of 25 μL. The original protocols were modified as follow: BstNI was substituted with 30 U of the more thermo stable PspGI restriction endonuclease (EuroClone, Pero, Milano, Italy), and the reverse primer (DIA) of the K-RAS codon 12 was changed to avoid the amplification of the K-RAS pseudogene (the new sequence is TTTACCTCTATTGTTGGATCATATTC). The amplified samples were visualized by electrophoresis through a Criterion Tris-HCl precast 10% polyacrylamide gel (Biorad, Segrate, Milano, Italy). The results were interpreted as reported by Mixich et al. [17]. The reverse DIA primer changed the size of the expected K-RAS mutated amplicons from 82 to 107 bp.

### Mutational analysis of the K-RAS and BRAF genes by Pyrosequencing

K-RAS codons 12 and 13 or BRAF codon 600 mutations were detected by Pyrosequencing [18] in mCRC or mMEL samples according to the manufacturer's instructions (Diatech Pharmacogenetics, Jesi, Italy). Briefly, DNA from patients and controls were amplified in a Rotor-Gene Q 2plex HRM instrument (Qiagen, Manchester, United Kingdom) by specific PCR protocols (Anti-EGFR MoAb Response-KRAS status or Anti-EGFR MoAb Response-BRAF status). The endpoint PCR products were sequenced in a PYROMARK Q96 instrument and analyzed according to the manufacturer's instructions.

### Mutational analysis of the K-RAS, BRAF and EGFR genes by Sanger Sequencing

The Sanger Sequencing method [19] was applied to samples with doubtful K-RAS and BRAF analyses after Pyrosequencing, and as reference test for the analysis of mutations at the exons 18–21 of the EGFR gene.

To amplify K-RAS or BRAF, 70 ng of genomic DNA were mixed in 50 μL to 1x buffer, 1.5 mM of MgCl_2_, 200 μM of dNTP, 1.2 U of AmpliTaq Gold DNA Polymerase (Invitrogen, Carlsbad, CA) and 0.5 μM of primers. The K-RAS primers (GTACTGGTGGAGTATTTGATAGTGT and TTTACCTCTATTGTTGGATCATATTC) were designed using the software Primer3 (*http://frodo.wi.mit.edu/primer3/*) on the human K-RAS genomic sequence (*http://www.ncbi.nlm.nih.gov/gene* *) (ID*:*3845*) and produced amplicons 212 bp long. The BRAF primers were derived from Moroni et al. [20], generating amplicons of 228 bp.

To amplify exons 18–21 from EGFR, 35 ng of genomic DNA were mixed in 25 μL with the same reagents previously described, except for the substitution of the AmpliTaq Gold DNA Polymerase with 0.5 U of Platinum Taq DNA Polymerase (Invitrogen, Carlsbad, CA). The primer concentrations were 0.4 μM, and their sequences were defined in accordance with Lynch TJ et al. [21]. The exon-specific PCR products were 400, 372, 408 and 415 bp long.

PCR conditions consisted of a unique touchdown scheme (an annealing decrease of 3°C each 3 cycles starting from 64°C up to 57°C) that was repeated for 44 cycles.

The PCR products were then purified following the manufacturer's instructions (AMPure PCR Purification Kit protocol, Agencourt, Beckman Coulter, Brea, CA, USA) and submitted to cycle sequencing reactions (BigDye Terminator v1.1 Sequencing kit, Applied Biosystems, Foster City, CA, USA) in the presence of 0.1 μM of forward and reverse primers (as well as for the PCRs), separately. Capillary electrophoresis of purified sequences (CleanSeq System kit, Agencourt, Beckman Coulter, Brea, CA, USA) was performed at 60°C through a POP-7 polymer in a 3130 four-capillary sequencer (Applied Biosystems, Foster City, CA, USA). The Sequencing platform (Applied Biosystems, Foster City, CA, USA) was used to acquire samples, which were then analyzed with the Variant Reporter software (Applied Biosystems, Foster City, CA, USA).

### Mutational analysis of the EGFR gene by Therascreen

The Therascreen EGFR RGQ PCR kit detects 29 somatic mutations frequently occurring within exons 18–21 of the EGFR gene. This PCR kit applies the Scorpions and ARMS technologies to a real-time PCR running in a Rotor-Gene Q 2plex HRM instrument (Qiagen, Manchester, United Kingdom).

PCR conditions and criteria of interpretation of the results were adopted according to the manufacturer's protocol (Qiagen, Manchester, United Kingdom). Briefly, samples with a cycle threshold (C_T_) of the HEX control signal ≥ 37 were rejected, and samples with a C_T_ of FAM mutation signal ≥ 40 were scored as negative or indeterminate, as they were below the sensitivity limit of the assay. To define EGFR positivity, specific ranges of acceptable ΔC_T_ values were defined in the manual.

### Sensitivities of the assays

Home-made reactions were designed to determine the sensitivities of the assays by using mutated DNA from specific cell lines progressively diluted in blood-derived DNA of a healthy donor. Briefly, the A549 cell line was homozygous for the K-RAS p.Gly12Ser mutation, the HT29 was heterozygous for the BRAF p.Val600Glu mutation, the H1650 was heterozygous for the EGFR deletion at the exon 19 (p.Glu746_Ala750del), and the H1975 was heterozygous for the EGFR p.Leu858Arg mutation at the exon 21. The cell lines were purchased from the ATCC (LGC Standards S.r.l., Sesto San Giovanni, Milan, Italy). K-RAS mutations were detected by REMS-PCR, Pyrosequencing and Sanger Sequencing. Their sensitivities for heterozygous mutations were near 0.1%, 5% and 20%, respectively. BRAF mutations were detected by Pyrosequencing or Sanger Sequencing at sensitivities near 5%. EGFR mutations were detected by Sanger Sequencing up to 6% of sensitivity (data not shown). As reported (Therascreen protocol, Qiagen, Manchester, United Kingdom), the sensitivities of the Therascreen assay for the EGFR mutations ranged from 0.5% (p.Leu861Gln) to 7.02% (p.Thr79Met).

### External Quality Assurance (EQA)

As reference laboratory for the diagnostic assessment of mutational screening in solid tumors since 2010, we have been participants in several EQA schemes of the European Society of Pathology (ESP) for K-RAS (four times) and EGFR (one time) mutations and of the AIOM-Italian Society of Pathological Anatomy and Cytology schemes (SIAPEC) for K-RAS (one time), BRAF (one time) and EGFR (two times). We successfully passed all the EQA procedures (data not shown).

### Statistics

Pearson's Chi square test (with the application of a Yates's correction when required) or Fisher's exact test were alternatively applied to Contingency 2x2 Tables comparing mutation frequencies or tissue characteristics in samples that were classified acceptable and not acceptable on the basis of morphologically defined tumor cell contents (*www.openepi.com*). The Chi square P value was calculated on a 2x3 Contingency Table to compare the frequency of mutation variants in mutated samples belonging to different categories of patients. Significance was set at P<0.05.

## Results

### Frequency of *NA* specimens

From January 2010 to August 2013, 1797 FFPE tumor samples, derived from patients with mCRC, mMEL and NSCLC were selected to detect K-RAS, BRAF and EGFR somatic mutations, respectively. All samples were submitted to DNA sequence analyses irrespective of their *A* or *NA* status.

First, we compared the frequency of *NA* in the mCRC and NSCLC specimens that were analyzed with different morphological criteria of unsuitability (in 2010, the tumor cell cut-off was 70%, but from 2011–2013 the tumor cell cut-off was 50%). The ratio for *NA* tumor specimens was 10/332 and 41/752 (3.0% *vs* 5.4%) in mCRC samples and 15/74 and 106/498 (21.3% *vs* 20.3%) in NSCL tumor specimens within the 2010 and 2011–2013 periods, respectively. The difference was not statistically significant (p>0.05). Therefore, we evaluated as a single cohort the specimens and the results collected from 2010–2013.

**Fig 1:**
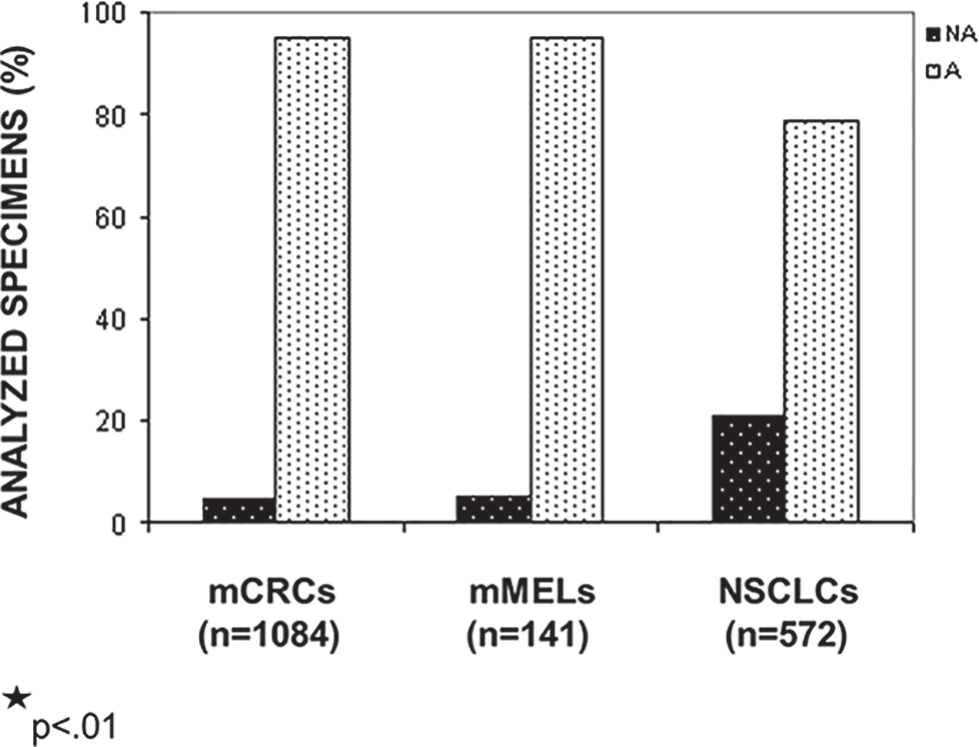
Morphologically acceptable and not acceptable tumor specimens in FFPE tissues from mCRC, mMEL and NSCLC patients. The frequencies of *NA* specimens were significantly higher in the NSCLC patient group (p<0.01). *X-axis*: pathological categories divided in *NA* and *A* specimens; *y-axis*: percentage of the analyzed specimens.

As shown in Fig 1, the *NA* tumor specimens were significantly more frequent (121/572) (21.1%) in NSCLCs than in mCRCs (51/1084) (4.7%) and in mMELs (7/141) (5.0%) (p<0.01).

### Frequency of *NA* specimens in cytological and histological tumor samples

DNA was extracted from 190 (10.6%) cell blocks (from cytological sampling) and 1607 (89.4%) tissue blocks (biopsy or surgical specimens). Cytological specimens represented 1.3% of samples in mCRCs, 2.1% in mMELs and 30.2% in NSCLCs (p<0.0001). The frequencies of *NA* specimens in cytological and histological samples were 14.3% *vs* 4.6% in mCRCs, 33.3% *vs* 4.3% in mMELs and 35.3% *vs* 15.0% in NSCL tumors (Table 1), respectively. The frequency of the *NA* samples was significantly higher in cytological specimens of NSCLC (p<0.01).

**Table 1:** Frequencies of acceptable and not acceptable tumor samples in cytological and histological specimens from mCRC, mMEL and NSCLC patients.

		CYTOLOGICAL SPECIMENS (n = 190)		HISTOLOGICAL SPECIMENS (n = 1607)	
		n	(%)	n	(%)
**mCRCs (n = 1084)**	***A***	12	(85.7)	1021	(95.4)
	***NA***	2	(14.3)	49	(4.6)
**mMELs (n = 141)**	***A***	2	(66.7)	132	(95.7)
	***NA***	1	(33.3)	6	(4.3)
**NSLCs** * **(n = 572)**	***A***	112	(64.7)	339	(85.0)
	***NA***	61	(35.3)	60	(15.0)

*A*:Acceptable samples

*NA*:Not acceptable samples

*p<0.01

### Distribution of wild-type and mutated K-RAS gene sequences among *NA* and *A* specimens from mCRC patients

The main purpose of this work was to establish if *NA* and *A* specimens, derived from stringent criteria of microscopically revision of tissue sections, corresponded to a statistically significant different number of wild-type (WT) and mutated (MUT) samples for the genes analyzed. As the analytical sensitivities were different for each sequenced gene (see *materials and methods*), we separately calculated the frequencies of WT and MUT samples in *NA* and *A* specimens belonging to the three different diseases.

**Fig 2:**
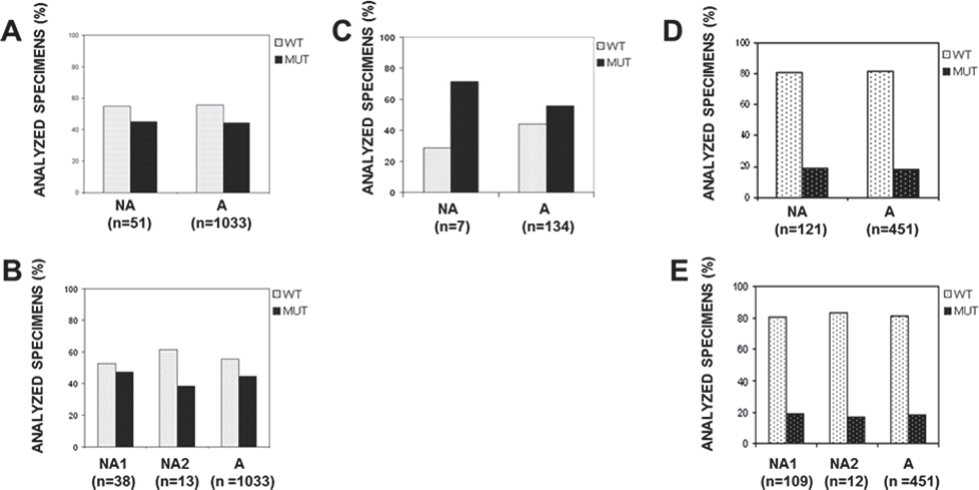
Distribution of specific somatic mutations among morphologically acceptable and not acceptable tumor specimens. Acceptable (*A*) and not acceptable (*NA*) tumor specimens with mutated (MUT) or wild-type (WT) DNA sequences. (**A**) Codons 12 and 13 of the K-RAS in mCRC patients. (**C)** Codon 600 of the BRAF in mMEL patients. (**D**) Exons 18–21 of EGFR in NSCLC patients. Mutated or wild-type NA tumor specimens at K-RAS (**B**) and EGFR (**E**) were further divided into samples with less than 50% (70% before 2011) enriched tumor cells (*NA1*) and samples with less than 100 tumor cells (*NA2*) per FFPE tissue section. Different frequencies of mutated/wild-type specimens between *NA* (*NA1* and *NA2*) and *A* samples did not result statistically significant (p>0.05). X-axis: categories of morphological suitability; y-axis: percent of samples with wild-type or mutated sequences.

Fig 2A shows the frequencies of WT and MUT samples in *NA* and *A* specimens of mCRC patients. The number of WT and MUT specimens were 28 and 23 out of 51 (54.9% and 45.1%, respectively) in the *NA* samples and 575 and 458 out of 1033 (55.7% and 44.3%, respectively) in the *A* samples. In *NA1* samples WT and MUT specimens were 20 and 18 out of 38 (52.6% and 47.4%, respectively) and in *NA2* samples 8 and 5 out of 13 (61.5% and 38.5%, respectively) (Fig 2B). The *NA2* group showed a higher percentage (not statistically significant) of WT specimens than both the *NA1* or the *A* group.

Three of the *NA* samples had a second acceptable (*A*) specimen available. One of these cases, which contained less than 10% tumor cells and resulted in WT K-RAS, was MUT in the *A* specimen.

### Distribution of wild-type and mutated BRAF gene sequences among *NA* and *A* specimens from mMEL patients

As shown in Fig 2C, in the 7 *NA* samples the number of WT and MUT specimens was 2 and 5 (28.6% and 71.4%, respectively), and in the 134 *A* samples the WT and MUT distribution was 59 and 75 (44.0% and 56.0%, respectively). In the *NA1* samples the number of WT and MUT specimens was 1 and 4 out of 5 (20.0% and 80.0%, respectively) and 1 and 1 out of 2 (50.0% and 50,0%, respectively) in the *NA2* (data not shown) (differences were not statistically significant).

### Distribution of wild-type and mutated EGFR gene sequences among *NA* and *A* specimens from NSCL adenocarcinoma patients

In the 121 *NA* samples, the number of WT and MUT specimens was 98 and 23 (81.0% and 19.0%, respectively) and in 451 *A* samples was 369 and 82 (81.8% and 18.2%, respectively) (Fig 2D). The number of WT and MUT specimens was 88 and 21 out of 109 (80.7% and 19.3%, respectively) in the *NA1* samples and 10 and 2 out of 12 (83.3% and 16.7%, respectively) in the *NA2* samples (Fig 2E) (all differences statistically not significant). In 8 *NA* samples, two of which (with less than 5% and 100 tumor cells) EGFR MUT, the analysis performed in a second *A* specimen confirmed the 2 MUT and the 6 WT (data not shown).

### Distribution of K-RAS and EGFR mutations between *NA* and *A* samples compared to results collected from published datasets

We measured the frequency of the principal mutation variants occurring at the codon 12 of K-RAS and at the exons 18, 19, 20 and 21 of EGFR among *NA* and *A* samples and compared the results to specific online databases, as reported below.

**Fig 3:**
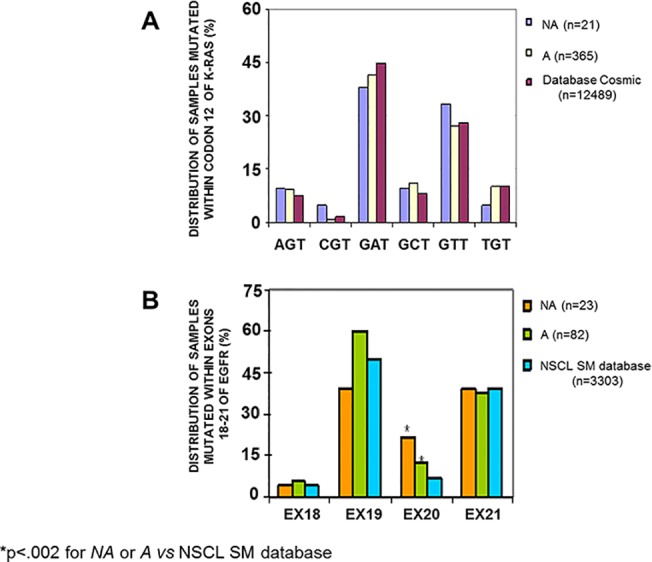
Specific somatic mutation frequencies in samples morphologically acceptable, not-acceptable and derived from published data. The frequencies of each specific mutation at codon 12 of K-RAS in not acceptable (*NA*) or acceptable (*A*) mCRC tumor specimens compared to the corresponding online dataset (**A**) were not statistically different (p>.05). Mutation frequencies at the exons 18–21 of EGFR were statistically different only for the exon 20 between *NA* or *A* specimens versus the online dataset (**B**). X-axis: specific K-RAS or EGFR mutations, divided into *NA*, *A* and the online dataset samples; Y-axis: frequency distributions of mutated samples.

The mutation frequencies related to codon 12 of K-RAS within our dataset were compared to the Cosmic database (Sanger Institute database; *www.sanger.ac.uk/genetics/CGP/cosmic/*) and were calculated as number of specimens with each specific mutation divided by the totality of the mutated specimens (Cosmic n = 12489, *NA* n = 21 and *A* n = 365). The frequencies are shown in Fig 3A and reported in Table 2. To obtain a Chi square P value, a 2x3 Contingency Table was applied to compare the frequency of each mutation variant (measured on the total mutations) in the three groups, with the exception of the "CGT" mutated samples, which did not meet the Cochrane recommendations [22] and to which a Fisher's exact test was applied in a classical 2x2 Contingency Table. The mutation frequencies analyzed within the *NA*, *A* and database samples did not result in statistically significant differences (Fig 3A).

**Table 2:** Frequency distribution of single point mutations occurring at codon 12 of K-RAS in morphologically not acceptable, acceptable and dataset mCRC-derived tumor specimens.

	MUTATED VARIANTS AT CODON 12 OF K-RAS					
MUTATED mCRC SAMPLES	AGT	CGT	GAT	GCT	GTT	TGT
***NA* (n = 21)**	2	1	8	2	7	1
***A* (n = 365)**	34	3	152	40	99	37
**COSMIC DB (n = 12489)**	930	193	5582	1002	3499	1283

*NA* = Not acceptable samples

*A* = Acceptable samples

Cosmic DB = Cosmic database of somatic mutations

The distribution of the EGFR gene mutations occurring at the exons 18, 19, 20 and 21 in the *NA*, *A* and online dataset samples (obtained from the NSCL SM-EGFR-database, *www.somaticmutations-egfr.org/overall_mutation_frequency.html*) is shown in Fig 3B and reported in Table 3. Because the EGFR mutations were too complex for detailed analysis, we grouped them on the basis of the exons involved. The frequency was calculated as the number of specimens with each exon-specific mutation, divided for the sum of the mutated specimens (SM-EGFR-database: 3303 cases, *NA*:23 cases and *A*:82 cases). In some samples from our dataset, multiple coexisting mutations were found. The different frequencies of samples with each mutation among the three categories were not statistically significant, except for exon 20, which was highly mutated in both the *NA* and *A* specimens compared to the online dataset group (p<0.002) (Fig 3B).

**Table 3:** Frequency distributions of mutations within exons 18–21 of EGFR in morphologically not acceptable, acceptable and dataset NSCLC-derived tumor specimens.

	MUTATED VARIANTS OF EGFR			
MUTATED NSCLC SAMPLES	EXON 18	EXON 19	EXON 20	EXON 21
***NA* (n = 23)**	1	9	5	9
***A* (n = 82)**	5	49	10	31
**NSCL SM-EGFR DB (n = 3303)**	137	1662	213	1291

*NA* = Not acceptable samples

*A* = Acceptable samples

NSCL SM-EGFR DB = Database of EGFR somatic mutations from NSCL cancer patients

## Discussion

In the present work we demonstrated that the frequency of mutated and wild-type DNA sequences in hotspot regions of K-RAS, EGFR and BRAF genes were poorly affected by the current definition of cell or tissue sample suitability. In particular, by analyzing the frequencies of activating mutations and wild-type gene sequences in a total number of 179 *NA* and 1618 *A* specimens grouped for tumor pathology, we demonstrated that: 1) the cytological specimens derived from bronco-aspirate in the NSCLC set were more frequently *NA* due to hypocellularity; 2) the proportion of *NA* specimens is not modified by lowering the cut-off from 70% to 50%; 3) the frequencies of WT and MUT sequences were similar between *NA* and *A* specimens in every patient group, and neither the percentages nor the number of tumor cells present in the samples affected the WT frequency; and 4) the frequency of mutation variants occurring at specific hotspots (codon 12 of K-RAS and exons 18 to 21 of EGFR) was equivalent in *NA* and *A* samples.

Focusing on the *NA1* category, which refers to the percentage of tumor cells, the cut-off value of 70% of tumor cells per section, than lowered to 50%, was originally set based on the sensitivity of the direct sequencing, generally reaching 25%-30% to detect homozygous mutation pattern (50–60% for heterozygous genotypes) [23, 24]. In the present study, we could not establish an appropriate lab-tailored cut-off, because the percentage of tumor cells present in each sample was reported as within or below the cut-off and not as defined number. Nevertheless, when the appropriate size of *NA* specimens in each pathological patient group is achieved, it can be subdivided in five categories of decreasing tumor enrichment (from 50% to 0%). Therefore, the distribution of gene mutations between each category and the *A* series becomes comparable. Lab-tailored cut-offs will then be comprised in the last category of tumor enrichment behaving as the adequate samples. Although our lab-tailored cut-off was not yet determined in the present study, mutated specimens in the *NA1* series most likely contain a percentage of mutant DNA cells higher than 20%, corresponding to the minimal rate detectable by our less sensitive assay (Sanger Sequencing) applied to tumor DNA heterozygous for the examined mutation. New generation assays with higher sensitivity further reduces the need for high percentages of tumor cells.

The cut-off issue is further complicated by the occurrence of intrinsic tumor heterogeneity. In this regard, Yu and co-authors [10] demonstrated that the percentage of K-RAS mutant DNA in enriched tumor areas from FFPE blocks of 47 mCRC showed a continuous distribution from 10.8% (high heterogeneity) to 98.3% (low heterogeneity). Assuming, for example, a sensitivity limit of 5% to detect K-RAS mutations by Pyrosequencing, the starting percentage of morphologically enriched tumor cells should vary between 46.3% and 5.1% for tumors highly or barely heterogeneous for the tested mutation, respectively. Thus, the tumor enrichment required before molecular biology testing should be directly proportional to the tumor heterogeneity for mutant alleles and inversely proportional to the assay sensitivities. Since tumor heterogeneity varies among patient samples, histology-driven tissue multisampling combined with cell pooling and cell sorting techniques should be applied to a large series of specimens upstream, in order to define the best morphological cut-off in relation to both the extent of tumor heterogeneity and the specific analytical methods used for mutational testing. We cannot exclude the possibility that in our series few *NA* specimens presented tumor heterogeneity or whenever homogeneous for the mutations analyzed, their tumor abundance was below the detection level of the assays. Nevertheless, our data suggest that the number of these specimens was low enough to maintain unchanged the overall results. One example is the *NA* mCRC specimen, which resulted in a false WT when compared with an *A* sample derived from the same patient three years later. In this case, tumor heterogeneity or clonal evolution [25] cannot be excluded. Conversely, in the NSCLC group one *NA1* specimen coupled with its *A* counterpart was truly mutated, although the former contained less than 5% enriched tumor cells, in line with the sensitivity cut-off level of the assay used and with a presumed complete homogeneity of cancer cells.

About the *NA2* cut-off, the amount of genomic DNA corresponding to 100 diploid tumor cells in G1 phase is comprised between 500 and 1000 pg, as one cell contains near to 7 pg of DNA [26]. This range is slightly below the optimal input of good quality DNA suggested to obtain PCR products reliable for forensic genotyping [27]. The results obtained in the present study show that the collection of more tissue sections in specimens defined as *NA2* (less than 100 tumor cells per section) may be a good solution for recovering a sample suitability for molecular analysis.

Finally, we considered the test sensitivity, which may highly impact on the overall results, showing equivalent distributions of the mutation variants between *NA* and *A* samples. A significantly higher percentage of NSCLCs that contain a mutated exon 20 in EGFR was found in our series when compared to the specimens belonging to the lung database collection. Although we cannot exclude the hypothesis of different assay sensitivities for that mutation, we have also considered a bias in the data collection between our series and the published results, or the coexistence of multiple mutations as demonstrated in some specimens of our series, which may have been less prevalent or detectable in the online lung dataset.

In conclusion, the molecular methods recently introduced in diagnostic practice are more sensitive than in the past. These new procedures may need less strict pre-analytical purification of the tumor tissue areas in order to identify the attended mutations. Our data demonstrate that the frequency of mutated or wild-type specimens is identical in samples within and below the international cut-offs of suitability. This observation suggests extending the DNA sequencing analyses also to the *NA* specimens, thus leaving the final definition of true positive (mutated) or putative false negative (wild-type) cases to the "post-analytical" phase. New and more flexible lab-tailored cut-offs need to be considered, depending on the molecular biology methods used and the extent of the tumor heterogeneity.
